# Artificial Intelligence Tools for Refining Lung Cancer Screening

**DOI:** 10.3390/jcm9123860

**Published:** 2020-11-27

**Authors:** J. Luis Espinoza, Le Thanh Dong

**Affiliations:** 1 Global Health Unit, Faculty of Health Sciences, Kanazawa University, Kanazawa 920-0942, Ishikawa, Japan; 2 Department of Hematology and Rheumatology, Faculty of Medicine, Kindai University, Osaka-Sayama 589-8511, Osaka, Japan; 3 Center for Gene and Protein Research, Faculty of Medical Technology, Hanoi Medical University, Hanoi 100000, Vietnam; dongle.freelancer@gmail.com

**Keywords:** lung cancer screening, early cancer diagnosis, lung cancer imaging, artificial intelligence and lung cancer, computers assisted diagnosis

## Abstract

Nearly one-quarter of all cancer deaths worldwide are due to lung cancer, making this disease the leading cause of cancer death among both men and women. The most important determinant of survival in lung cancer is the disease stage at diagnosis, thus developing an effective screening method for early diagnosis has been a long-term goal in lung cancer care. In the last decade, and based on the results of large clinical trials, lung cancer screening programs using low-dose computer tomography (LDCT) in high-risk individuals have been implemented in some clinical settings, however, this method has various limitations, especially a high false-positive rate which eventually results in a number of unnecessary diagnostic and therapeutic interventions among the screened subjects. By using complex algorithms and software, artificial intelligence (AI) is capable to emulate human cognition in the analysis, interpretation, and comprehension of complicated data and currently, it is being successfully applied in various healthcare settings. Taking advantage of the ability of AI to quantify information from images, and its superior capability in recognizing complex patterns in images compared to humans, AI has the potential to aid clinicians in the interpretation of LDCT images obtained in the setting of lung cancer screening. In the last decade, several AI models aimed to improve lung cancer detection have been reported. Some algorithms performed equal or even outperformed experienced radiologists in distinguishing benign from malign lung nodules and some of those models improved diagnostic accuracy and decreased the false-positive rate. Here, we discuss recent publications in which AI algorithms are utilized to assess chest computer tomography (CT) scans imaging obtaining in the setting of lung cancer screening.

## 1. Introduction

Lung cancer was first recognized as a distinct clinical entity in 1810 [1], and by the beginning of the 20th century, it remained a relatively unknown disease, with only 374 documented cases in the world [2]. However, the incidence of this disease increased dramatically following the broad introduction of cigarette smoking habits in human populations [3], and at present, lung cancer is the most frequent and deadly cancer worldwide with over two million new cases diagnosed per year, causing more cancer-related deaths than other common cancers including colon, breast, and prostate cancers combined [4].

Etiologically, more than 85% of lung cancers are caused by long-term tobacco smoking and the remaining cases, diagnosed in never-smoked individuals, are attributed to a combination of factors, including genetics and exposure to other carcinogens such as radon gas, asbestos, second-hand smoke, and other forms of air pollution [4]. 

Despite the fact that significant advances in the diagnosis and treatment of lung cancer have been made, the disease is still associated with poor clinical outcomes [5,6] and survival is strongly determined by the stage of disease at diagnosis and thus, whereas the five-year survival rate for patients with the early-stage disease is 56%, in those with advanced disease the 5-year survival rate is less than 5% [2]. Considering that only 16% of lung cancers are diagnosed in the early stage and that most patients present with advanced disease [7,8], developing screening tests capable of detecting the disease in the early stages has been a long-term goal in lung cancer care. Several screening methods have been tested so far, including sputum cytology, chest radiographs (CXR) [9], and low-dose computer tomography (LDCT), and recently the analysis of various biomarkers, however, data from clinical trials indicate that only the use of low-dose computer tomography scans (LDCT) in heavy smoker individuals has been associated with a significant reduction in lung-cancer-related mortality [10,11].

Although the introduction of targeted therapies and immunotherapeutic agents, especially immune checkpoint inhibitors (utilized alone or in combination with standard chemotherapeutic regimens), have resulted in a longer duration of overall survival compared with standard chemotherapy [12], these novel therapies are not effective in all patients; thus, early detection remains the most important intervention window for improving patient survival.

Despite the fact that screening lung cancer with LDCT has demonstrated a clear benefit for reducing all-cause mortality, the high rate of false-positives and the cost of unnecessary diagnostic procedures needed to confirm or rule out those false-positives are important limitations of this approach [11]. The emergence of artificial intelligence (AI) as a new tool for assessing medical data implies new opportunities for improving the diagnosis and treatment of various human diseases [13,14,15,16]. In the case of lung cancer diagnosis, coupling AI algorithms with available clinical and biomedical data has the potential to improve lung cancer screening methods [17]. For example, AI has the potential to improve the analysis and interpretation of lung images obtained by magnetic resonance images (MRI )or computer tomography (CT) scans and could be helpful to better decipher the clinical significance of data derived from tissue or fluid biomarkers, electronic medical record (EMR), and metagenomic data leading to improved diagnosis of lung diseases [18,19] and recent studies have utilized various AI algorithms to better interpret LDCT images in an attempt to refine lung cancer screening. In this article, we aimed to review publications in which artificial intelligence tools were utilized in the setting of lung cancer screening. For that purpose, we assessed studies that developed or validated machine learning or deep learning models for the early diagnosis of lung cancer from chest CT scans obtained in the setting of lung cancer screening. We searched Pubmed and the Cochrane Database for studies published from 1 January 2012, to 30 September 2020. A manual search of citations of included studies was also performed to identify any additional relevant articles that might have been missed by the searches. Articles reporting AI models to analyze CXR and MRI were not the focus of this review since these imaging modalities are not the main diagnostic tool utilized in lung cancer screening programs. Although there have been previous reviews on the utility of AI tools for cancer diagnosis, including lung cancer, this is the first in-depth review of the applications of AI algorithms for the analysis of chest CT scan images obtained in the setting of lung cancer screening.

## 2. Lung Cancer: Epidemiological and Clinical Considerations

Lung cancer typically occurs in older patients with a history of tobacco use (median age at diagnosis of 70 years) [20]. Non-smoking associated lung cancer appears to be a distinct entity that has been linked with specific genetic mutations, is relatively more frequent in women and Asians [21,22], and is also associated with exposure to certain environmental factors such as radon, asbestos, and other contaminants [4,6,23]. 

By the time of diagnosis, more than 50% of patients with lung cancer already have a metastatic disease, which is mainly due to the relative absence of specific symptoms during the early stages of the disease [3]. Indeed, lung cancer may be found incidentally on chest imaging in some patients. The most common symptoms at presentation are persistent cough or chest pain. Other symptoms include weight loss, hemoptysis, malaise, dyspnea, and hoarseness. In some patients, the disease presents with manifestations caused by distant metastases, such as compression involving the esophagus causing dysphagia, compression involving the laryngeal nerves causing hoarseness, or compression involving the superior vena cava causing facial edema and distension of the superficial veins of the head and neck. Infrequently, patients may present with a paraneoplastic syndrome such as hypertrophic osteoarthropathy with digital clubbing or hypercalcemia from parathyroid hormone-related protein. 

Based on histological and clinical features, two groups of lung cancers are clearly distinguishable: non-small-cell lung cancer (NSCLC) and small-cell lung cancer (SCLC). NSCLC accounts for over 80% of all lung cancers, being the major cause of lung cancer-related death worldwide and is classified as adenocarcinoma, squamous cell carcinoma, and large-cell lung cancer. Adenocarcinoma often occurs in the more peripheral regions of the lung and is mainly associated with tobacco smoking, although it can occur in nonsmokers. Squamous cell carcinoma tends to arise in the more central regions of the lungs and is also associated with tobacco smoking. Large cell carcinoma is often a rapidly growing tumor type that usually originates from neuroendocrine cells of the lungs and is the less common type among NSCLC. 

SCLC accounts for about 10 to 15% of all lung cancers and like NSCLC, it is also linked to tobacco smoking. Compared to NSCLC, these tumors are more aggressive and tend to metastasize early, often presenting with brain metastases [24], thus in general, surgery has limited application in SCLC, although lobectomy is recommended in patients with stage I without the involvement of mediastinal and supraclavicular regions [25]. Moreover, although some clinical response can be observed after first-line treatment, the majority of patients ultimately die of disease relapse [25,26]. 

The discovery of genetic mutations such as c-Myc, c-Met, Bcl-2, p53, Rb, and other DNA molecular changes associated with lung cancer led to the development of targeted therapies with the potential to improve anti-tumor efficacy while decreasing toxicity [27], however, for most patients with lung cancer these agents did not meet the clinical expectations [28], illustrating the molecular complexity of the disease and the need of a more personalized approach to further optimize treatment [29]. Nevertheless, the development of immune checkpoint inhibitors, including anti-PD-1 and anti-PD-L1 monoclonal antibodies has revolutionized the treatment of lung cancer [30,31], and patients treated with these agents, either as monotherapy or in combination with chemotherapy or radiotherapy, exhibit better clinical outcomes including a superior progression-free survival (PFS), and improved overall survival, as well as lower adverse effects compared with patients treated with standard chemotherapy [32,33,34,35].

## 3. Lung Cancer Screening 

Cancer screening refers to the presumptive identification of undiagnosed cancer in asymptomatic individuals by performing tests, examinations, or other detection procedures that can be readily applied to the target population [36]. The goal of cancer screening is early disease detection, which can be translated into the design of more effective therapeutic interventions and ultimately a reduction in mortality. Examples of cancer screening methods with probed clinical utility include the use of mammograms and Papanicolaou cytology for the early diagnosis of breast cancer and cervical cancer respectively [37]. 

In the case of lung cancer, attempts to develop an effective screening method date back to the 1960s when the utility of CXR was investigated in clinical trials involving asymptomatic individuals in high-risk populations. These studies found that CXR did not significantly reduce the mortality from lung cancer in the populations at risk [38]. Similarly, the use of sputum cytology with or without CXR failed to show benefits in subsequent randomized trials conducted in the USA and Europe throughout the 1970s and the 1990s [39,40]. 

The introduction of CT technology in the clinical practice attracted the interest of clinicians for utilizing this imaging modality in early lung cancer diagnosis, however, due to the relatively high radiation exposure (7 millisieverts (mSv)) and the prolonged scanning time associated with conventional CT scans limited its application. The discovery that LDCT (radiation exposure of 1.6 mSv) could generate high-resolution images with similar sensitivity and specificity to conventional CT scans for lung nodule detection opened the way for the use of this technology in lung cancer screening. Early studies conducted in Japan suggested the feasibility of LDCT as a tool for early lung cancer detection [41,42]. This was further confirmed by the Early Lung Cancer Action Project (ELCAP) trial conducted in the United States, which showed that LDCT was superior to CXR to detect malignant and benign nodules (2.7% vs. 0.7% and 20.6% vs. 6.1%, respectively) [43]. An expanded version, the International-ELCAP (I-ELCAP), that included 38 centers in five countries with 31,567 screened patients, showed a 13% positivity in the initial LDCT scans and 5% in the subsequent annual scans. Lung cancer was detected in 484 patients (85% in stage I), and the estimated 10-year survival rate of patients with stage I lung cancer who underwent surgery was 92% [44]. Similarly, the Mayo Clinic LDCT study that prospectively enrolled 1520 asymptomatic current or former smokers who underwent baseline LDCT scans followed by annual LDCT screening found that among the 68 lung cancers detected 61% were stage I. Notably, the vast majority of nodules detected (95%) were found to be benign on follow-up [45].

The National Lung Screening Trial (NLST) was a large trial that began in 2002 and compared the effectiveness of LDCT scan versus CXR for lung cancer screening. A total of 3454 current (more than 30-pack-year history) or previous heavy smokers (less than 15 years since cessation) were randomized to receive either an LDCT scan or CXR annually for 3 years and were then followed for an additional 3.5 years with no screening. The study concluded that LDCT was associated with a significant 20% reduction in overall mortality among high-risk current and former smokers [46]. 

In the Danish Lung Cancer Screening Trial (DLCST), 4104 healthy heavy smokers/former smokers were randomized to five annual LDCT screenings or no screening. Lung cancer was more frequently diagnosed in the screening group (69 vs. 24) with more low stage tumors in the screening group than in controls (48 vs. 21). The authors concluded that CT screening for lung cancer detects more cancers and early disease, but does not significantly reduce mortality due to lung cancer [47]. Negative results in terms of reduction of lung cancer mortality were also reported in the Randomized Study on Lung Cancer Screening with Low-Dose Spiral Computed Tomography (DANTE) trial conducted in Italy, which the authors attributed to the limited statistical power of the study [48]. 

The ITALUNG study from Italy in which 3206 participants were randomized to LDCT (1613 subjects) versus no screening published their 4-year results. At the end of the follow-up period 67 lung cancer cases were diagnosed in the screening arm and 71 in the control arm with non-significant reductions of 17% (risk ratio (RR) = 0.83; 95% confidence interval (CI) 0.67 to 1.03) for overall mortality and 30% (RR = 0.70; 95% CI 0.47 to 1.03) for lung cancer-specific mortality. The authors concluded that despite the lack of statistical significance, outcomes of this trial suggest that LDCT screening could reduce lung cancer and overall mortality [49]. 

The German Lung cancer Screening Intervention (LUSI) is a randomized study that in investigated the role of LDCT screening in reducing lung cancer mortality in high-risk individuals and 8.8 years after randomization confirmed a lower hazard ratio for lung cancer mortality among those receiving LDCT screening compared with controls (0.74 95% CI: 0.46–1.19), with a stronger reduction of lung cancer mortality among women as compared to men [50].

More recently, the Multicentric Italian Lung Detection (MILD) trial evaluated the benefit of prolonged LDCT screening beyond 5 years, and its impact on overall and LC specific mortality at 10 years. A total of 4099 participants were prospectively randomized to a control arm (*n* = 1723) without intervention and a screening arm (*n* = 2376), which was further divided into annual (*n* = 1190) or biennial (*n* = 1186) LDCT for a median period of 6 years. The LDCT arm showed a 39% reduced risk of LC mortality at 10 years, compared with the control arm, and a 20% reduction of overall mortality indicating that prolonged LDCT screening (beyond five years) with biennial LDCT can achieve a reduction in lung cancer mortality that is comparable to that of annual LDCT [51,52]. In line with these observations, the Dutch Belgian Randomized Lung Cancer Screening trial (NELSON) randomized a total of 15,600 participants to undergo CT screening at baseline, year 1, year 3, and year 5.5 or no screening. At 10 years of follow-up, lung-cancer mortality was 2.50 deaths per 1000 person-years in the screening group and 3.30 deaths per 1000 person-years in the control group, which is an even bigger reduction in deaths from lung cancer than was seen in NLST [53].

As illustrated by the above studies, lung cancer screening with LDSCT has been extensively studied in the past decade, and some of these studies have shown promising results and has provided a rationale for the use of LDCT for lung cancer screening in high-risk ever-smokers. Indeed, the U.S. Preventive Services Task Force (USPSTF), in December 2013, endorsed the annual screening for lung cancer with LDCT as a preventive health service for the high-risk population (adults aged 55 to 80 years who have a 30 pack-year smoking history and currently smoke or have quit within the past 15 years) [54]. As more countries adopt this strategy for early lung cancer detection, it is worth it mentioning that this screening method is associated with various limitations, especially a high percentage of false-positives, which may result in unneeded treatment. Indeed, in the NLST, the vast majority of the pulmonary nodules identified in LDCT screens (96.4%) were not malignant [55]. In this regard, current criteria for distinguishing benign nodules from malignant ones are not well-established, thus, despite several efforts to address the limitations in lung cancer screening with LDCT, this technique frequently identifies a high proportion of pulmonary nodules that is not malignant. On the other hand, clinical and epidemiological studies have shown that a considerable proportion of newly diagnosed lung cancers were not covered by the NLST selection criteria [8,56], thus, there is a need for further complementary tests both to reduce the number of false-positives and to detect aggressive cancers early. 

Although public biomedical image benchmark databases have contributed to the development of image analysis algorithms, providing resources to evaluate, compare, and reproduce prior models, some datasets are distributed across multiple repositories or are indexed using different terminologies making it difficult to perform reliable comparisons and to promote reproducibility [57]. Bonafide benchmark of biomedical datasets with ground truth, such as the Lung Image Database Consortium image collection (LIDC-IDRI) have become available [58,59], which serve not only as a primary source for research purposes but also for the organization of image analysis challenges, where several teams compete to develop the best model for solving a given problem. In successful challenges relevant to lung cancer screening, such as the Lung Nodule Analysis 2016 (LUNA16) challenge and the Data Science Bowl 2017 prize, where the winning teams reported a high performance, teams utilized AI algorithms using annotated CT images from large public datasets to automatically and accurately diagnose lung lesions [60]. Although image analysis challenges play an important role in benchmarking algorithms for biomedical image analysis, they are associated with various deficiencies, including, heterogeneous design, lack of standards for challenge reporting, and inadequate interpretation and reproducibility of results [61]. To overcome these limitations, the BIAS statement (Biomedical Image Analysis Challenges) was recently proposed, which includes a checklist which authors of biomedical image analysis challenges are encouraged to include in their submission in an attempt to standardize and facilitate the review process and raise interpretability and reproducibility of challenges [62].

## 4. AI and CT Scan Images

LDCT is increasingly being adopted as a lung cancer screening method in various clinical settings, however, the accurate definition of a positive result and the management of lung nodules detected on LDCT scans are difficult challenges for the broad implementation of this method for lung cancer screening. Some of these issues could be addressed with the PanCan model, which estimates with high accuracy (area under the curve, AUCs of 0.94 in an external validation cohort) the malignancy probability of a pulmonary nodule detected in a baseline scan based on clinical data and nodule characteristics [63]; and in line with the PanCan model postulates, the Lung CT Reporting and Data System (Lung-RADS) was proposed by the American College of Radiology to estimate the malignancy probability of pulmonary nodules detected in baseline scans and based on risk assessment and imaging characteristics such as nodule type, size, solidity, and growth, to determine the appropriate patient follow-up strategy. Indeed, using Lung-RADS guidelines, various studies have shown a reduction of false-positive rates with the assessment of both baseline and subsequent scans [64,65]. 

Taking advantage of the unlimited capacity of computers for analyzing data and images, several studies have utilized AI tools, including machine learning, deep learning, and others, in an attempt to develop algorithms capable of identifying imaging features from LDCT images that may be specific to lung cancer, to more accurately differentiate between benign and cancerous nodules, which ultimately could help to improve lung cancer screening. Summarized clinical information of select studies is shown in Table 1 and relevant details of the reported algorithms are shown in Supplementary Table S1.

One of the first studies to illustrate the potential of AI algorithms to assist radiologists in the diagnosis of pulmonary nodules in the lung cancer screening setting utilized a deep learning system to analyze lung nodules with multi-stream convolutional network architecture and classifies nodule types relevant for patient management according to the Lung-RADS assessment categories and the PanCan malignancy postulates. The model was trained using data from the MILD trial (943 patients; 1352 nodules) and was independently validated using data from 468 patients (639 nodules) from the DLCST trial. Nodule segmentation was not required and the model was superior to patch classification of machine learning approaches and its performance was comparable, in terms of inter-observer variability, to four experienced radiologists [66]. 

Another study used a set of dynamic Bayesian networks to evaluate the utility of combining longitudinal data obtained during lung cancer screening programs to improve diagnostic accuracy. To train the models, the authors used LDCT screening outcome data, along with demographic information, smoking status, cancer history, family lung cancer history, exposure risk factors, comorbidities related to lung cancer from the LDCT arm of the NLST dataset, and further validated the models on the complete LDCT arm of the NLST dataset where it demonstrated satisfactory generalization, outperforming classical comparison models such as logistic regression and naïve Bayes [67], thus indicating that coupling LDCT imaging data with demographic and patients’ clinical characteristics may contribute to improving the accuracy of lung cancer screening programs. 

A three-dimensional convolutional neural network was employed to classify pulmonary nodules derived from clinical CT images. The model was first trained using LDCT images available in public databases obtained from lung cancer screenings and was validated using clinical LDCT images obtained from four different hospitals and was further evaluated, on a 50-image set where the patients underwent surgical dissection and had preoperative CT images prospectively collected where the performance of the algorithm was compared with that of 25 licensed physicians. The deep learning algorithm showed significantly better performance than manual assessment by trained physicians in detecting and classifying pulmonary nodules [68]. It must be noted that the validation data was derived from a multicenter dataset with variable image quality and the inclusion of a limited number of ground-glass nodules representing early-stage disease, which may have affected the nodule classification in this study, however, the fact that the model overperformed physicians assessing clinical images was an encouraging finding of this study further illustrating the feasibility of using deep learning algorithms for lung cancer screening in clinical practice.

Multiple machine learning-based methods were also employed to establish a framework for learning a partially-observable Markov decision process that simultaneously optimizes lung cancer detection while enhancing test specificity. The model was trained and tested using a dataset of 5402 single nodule unique trajectories of lung cancer screening patients from the NLST LDCT trial and used inverse reinforcement learning to discover a rewards function based on experts’ decisions. The model achieved a high accuracy with a true positive rate comparable to human experts while decreasing the false-positive rate [69]. 

Another study proposed a deep learning algorithm to predict the presence of lung cancer within a 3-year period evaluating all relevant nodule and non-nodule features on screening chest CT scans. The model was trained using data from the NLST trial, including participants who had received at least two CT screening scans up to 2 years apart, and was validated using data from participants in the PanCan study. This double validation was carried out by two groups of skilled chest radiologists in large academic centers assessing each LDCT image. The accuracy of the deep learning algorithm scores to predict lung cancer incidence at 1 year, 2 years, and 3 years was compared with that of the Lung-RADS system and volume doubling time, using the time-dependent area under the receiver operating characteristic curve (AUC) analysis. Compared with Lung-RADS, this model more accurately identified individuals at high risk and very low risk of developing lung cancer within 2 years [70]. The authors concluded this model could be used to accurately guide clinical management after the next scheduled repeat screening CT scan providing the framework to prospectively assess different screening intervals and more urgent diagnostic approaches for suspicious lung nodules based on malignancy risk.

Ardila and coworkers developed a three-dimensional deep convolutional neural network model using patients’ current and prior CT volumes from LDCT scans to predict the risk of lung cancer in individuals at high risk. When the model was tested against 6716 NLST cases it achieved a performance of 94.4% area under the curve (AUC) and when tested against an independent clinical validation set of 1139 cases, it performed similarly. Notably, when previous CT images were available, the model performed with similar accuracy with six expert radiologists, however, when prior CT images were not available, the model outperformed all six radiologists with absolute reductions of 11% in false-positives and 5% in false negatives [71]. It must be noted, however, that the radiologist-comparisons performed in this study were limited to retrospective data from the NLST dataset and clinical comparison metrics were limited to a small number of general radiologists. In addition, despite that the model appears to display consistent indicators of generalizability, the authors used only two datasets during testing, thus, although the model is promising, it needs to be further validated in other datasets and populations.

A recent study assessed the performance and effectiveness of deep neural networks for lung nodule detection by comparing the diagnostic efficacy of the model with that of radiologists evaluating real-world LDCT images. The model was trained with a large dataset of real-world clinical data (39,014 chest LDCT screening cases) and validated with a set of 600 cases and with CT images from the LUNA public dataset, demonstrated excellent performance in differentiating nodule dimensions and nodule sub-types. The model showed good agreement with radiology experts for detecting large and small lung nodules and was superior to expert radiologists in terms of identification sensitivity, showing also better receiver operating characteristic and area under the curve (ROC-AUC )performance and higher specificity than the average specificity of radiologists for classifying true positive cases [72]. Although certain baseline information, such as smoking history, lung diseases, and comorbidities were not available for this study, the fact that the model was trained with a large multi-center clinical database, is an important strength of this study and indicates the generalizability of applying this algorithm.

Radiomics is an emerging field that extracts multiple features from medical images and translates them into mineable data, which can be further used for the creation of statistical models and predictive analytics [73]. Radiomics can include several features of the objects to analyze including size, shape, texture, and can thus quantitatively and objectively define tumors and nodules. Because radiomics is applied to clinically available images, it can be also integrated with genomics, plasma biomarkers, biopsy staining patterns, and other patient-derived data. In addition, radiomics can be coupled with AI to take advantage of the superior capability of AI in handling a massive amount of data [74]. Indeed, both radiomics and AI are being utilized in the setting of radiological diagnosis of various diseases, including lung cancer [73]. 

For example, using public data from the NLST, one study extracted radiomic features of malignant nodules (196 patients) and benign pulmonary nodules (404 patients) to predict the subsequent emergence of cancer and based on 23 stable features in a random forests classifier the model could predict nodules that would become malignant one or two years with an accuracy of 80% [75]. Similar performance metrics (0.80 for the positive predictive value, 0.36 for the false-positive rate, and 0.80 for the area under the ROC curve) were reported in another study that localized thin-section CT images of 122 nodules and 374 radiomic features and integrated them with machine learning classification. Radiomic features, including CT density, sigma, uniformity, and entropy were useful in differentiating between benign and malignant nodules [76]. Various machine learning classifiers were also used to accurately predict lung cancer nodule status while also considering the associated false-positive rate by analyzing radiomic quantitative biomarkers taken from imaging data of lung nodules identified by LDCT scans. Imaging biomarkers (416 in total) were created from both nodule and parenchymal tissue from 200 patients and linear, nonlinear, and ensemble predictive classifying models were used to classify malignant or benign nodules. This model achieved a false-positive rate of 30%, which is significantly lower than that reported in the NLST, thus indicating that radiomics coupled with machine learning algorithms have the potential to provide good classification and simultaneously reduce the false-positive rate [77]. Finally, using data from two independent cohorts of patients with lung cancer (262 from North America and 89 from Europe) another study identified associations between radiomic imaging features, molecular pathways (immune response and inflammation), and clinical factors [78]. Importantly, in this model, prognostic biomarkers performed better when combining radiomic, genetic, and clinical information, thus indicating the complementary value of radiomics to integrate different characteristics of tumors. Therefore, radiomics, in combination with AI, may potentially enable practical use of precision medicine for the development of clinical biomarkers for diagnosis, prognosis, and prediction of outcomes and toxicity for individual patients of response to specific treatments.

## 5. Concluding Remarks and Future Directions 

Lung cancer is a largely preventable disease, with the majority of cases linked to tobacco smoking. Since most cases are diagnosed in an advanced stage, most patients have a poor prognosis after diagnosis. Failure of early diagnosis due to the lack of an effective screening test is therefore one of the major determinants for this dismal survival rate in lung cancer and constitutes an unmet clinical need in lung cancer care. Although the introduction of immunotherapy in recent years has dramatically improved survival rates in some patients with lung cancer compared with standard chemotherapy, even among patients with advanced disease, the high cost of these agents makes them almost prohibitive outside the developed countries. 

Large clinical trials have shown that lung cancer screening with LDCT results in better survival in high-risk populations compared with CXR but it associates with some drawbacks, particularly a high rate of false-positives as detailed above. With the transition from conventional biostatics to more sophisticated approaches like machine learning or deep learning for imaging analysis, it has become evident that AI could emerge as a formidable tool to refine the diagnosis of lung cancer. AI has the potential to increase the efficiency, reproducibility, and accuracy of tumor identification, not only via automated segmentation but also with the rapid expansion of computing speed and the increased efficiency of AI algorithms, it is likely that a separate segmentation analysis of suspicious images will be unnecessary making possible to evaluate whole-body imaging data with the help of AI algorithms. In this regard, whole-body approaches will also allow a more accurate analysis of organ features that may be distorted by the pathological processes but that are not apparent to human vision. This implies that AI algorithms could be applicable in several aspects of early lung cancer diagnosis, including the development of risk prediction tools for the accurate identification of high-risk individuals, the precise discrimination between malignant and nonmalignant nodules, and the integration of data from imaging studies with information derived from serological and tissue biomarkers studies. 

As mentioned above, several clinical trials conducted in the past showed the lack of utility of CXR as a tool for lung cancer screening, however, given the relatively broad accessibility and low cost of this imaging technique, several clinicians still consider that CXR may have some utility in early lung cancer detection. Indeed, over the last 10 years, various AI algorithms have been reported to improve the diagnostic precision of CXR in several chest conditions [79]. For example, the use of computer-aided detection (CAD) systems improved radiologists’ performance in several settings with a significant reduction in false-positive detection of lung nodules on CXRs [80,81,82]. In addition, deep convolutional neural network models outperformed physicians, including thoracic radiologists, in CXRs classification and nodule detection performance for malignant pulmonary nodules [83] and were superior to the average of human readers in terms of sensitivity, false-positive detection for detecting operable lung cancer with CXRs [84], which suggest that CXR coupled with AI may have some value in lung cancer screening. 

Positron emission tomography (PET scans) is a promising tool for reliable assessing malignant diseases, including lung cancer. In particular, PET with F-18 deoxyglucose coupled to CT scan (18F-FDG PET/CT) imaging is being used for accurately identifying lung nodules, although variable results have been reported with some studies showing higher sensitivity than conventional CT scans for lung cancer detection [47,48,50,51]. A recent mathematical model retrospectively assessing 18F-FDG PET/CT images identified several clinical and image features for the accurate identification of malignant pulmonary nodules and the diagnosis of lung cancer [85]. Although promising, these results require validation studies before considering the incorporation of PET imaging into large-scale screening programs. Further, the high cost and the restricted availability of FDG-PET are also important limitations. In addition, the accuracy of 18F-FDG PET/CT in diagnosing malignancy is impaired in populations with endemic infectious lung disease compared with nonendemic regions [86]. Of note, a study conducted in Switzerland developed an artificial neural network that achieved a sensitivity of 95.9% and 91.5% and a specificity of 98.1% and 94.2%, at standard dose and ultralow-dose PET 3.3%, respectively [87]. These results suggest that machine learning algorithms may aid fully automated lung cancer detection even at very low effective radiation doses of 0.11 mSv. Besides, further development of low-dose FDG-PET might improve the specificity of lung cancer screening and also open doors to other applications. 

MRI has superior soft-tissue definition compared with other clinically available imaging modalities and the introduction of imaging optimization sequences with the use of high-performance gradient systems, have contributed to improving the quality of MRI of the lungs, allowing the detection of nodules (>4 mm) with reasonable spatial resolution. Indeed, it is expected that MRI will reach a similar sensitivity and likely better specificity than LDCT in early cancer detection, which warrant its potential utility in lung cancer screening [88], as shown by two recent studies in which MRI performed similarly to LDCT in lung cancer screening with comparable life expectancy benefit and superior cost-effectiveness [89,90]. It is expected that recent MRI developments, such as the high field MR scanners and ultrashort echo time MR images, will contribute to improving image quality and the suitability of MRI in lung cancer screening programs [88]. In this regard, a recent study successfully developed machine learning methods, particularly recursive feature elimination and support vector machine, to accurately distinguish benign and malignant pulmonary lesions based on quantitative radiomic features of multiparametric MRI [91], thus indicating that AI-based integration of MRI imaging has tremendous potential for refining the role of MRI in lung cancer screening. 

Histopathological diagnosis is a key component of modern medicine and represents the definitive method for confirming the presence or absence of disease, being also essential in assessing disease grading and progression. Routine assessment of histopathological specimens is performed under light microscopy with several limitations inherent to the manual processing of images and the subjective visual assessment from pathologists, however, the introduction of supportive computational image analysis, including AI tools for image analysis, facilitated by the use of whole slide images (the digitalized counterparts of glass slides obtained via specialized scanning devices), has permitted the incorporation of additional quantitative assessments in histopathological studies, thus increasing diagnostic throughput [92]. For example, deep learning-based pathological image analysis can integrate multiple measurements and image analysis tasks, including segmentation, counting, and tissue classification, with practical applications in the diagnosis, subtyping, and staging of various tumor types, including lung cancer [93,94]. Deep learning algorithms have the potential to integrate multiple features from pathological images with those obtained from CT or MRI images and could have practical applications in the setting of lung cancer screening. 

Potential biomarkers derived from body fluid and tissues including, whole blood, plasma, sputum, bronchial lavage, urine, and biopsy specimens have been explored in the search for surrogate markers of lung cancer and various studies have shown that circulating tumor cells [95], autoantibodies [96], microRNAs [97], blood proteomic profiling [98], and exhaled breath biomarkers [99] are promising molecular candidates for early detection of lung cancer. 

**Figure 1:**
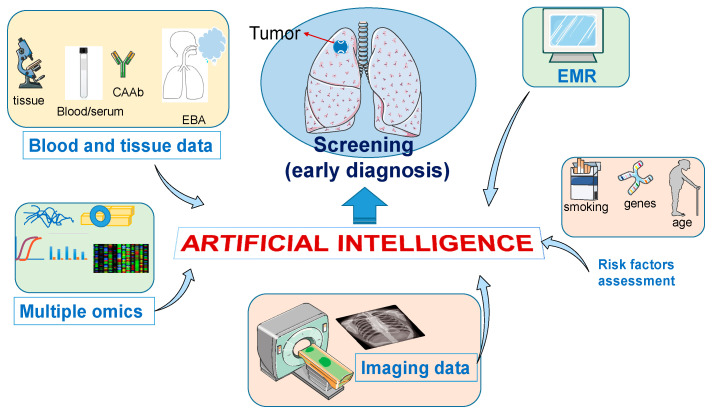
By integrating multiple data, AI algorithms have the potential to improve the early diagnosis of lung cancer. The utility of AI tools interpreting medical images has been demonstrated in several settings and in several diseases, including lung cancer. However, the usefulness of AI in contributing to the early diagnosis of lung cancer may extend beyond the interpretation of lung images. For example, blood samples are currently interrogated for the presence of circulating autoantibodies (CAAb), microRNAs, and various serum biomarkers, and some of these factors appear to be associated with lung cancer, thus AI could aid in the identification of specific patterns or signatures typical of lung cancer. Similarly, the presence of distinct patterns in exhaled breath analysis (EBA) are currently being studied for its utility in lung cancer diagnosis, and data from Electronic medical records (EMR), which constitutes a formidable source of clinical, demographic, and biometric data, coupled with AI algorithms could be a powerful diagnostic tool. Finally, high-throughput technologies such as metabolomics, transcriptomics, epigenomics, and the integration of multiple “omics” together with information from medical images and clinical data will provide insightful information for the understanding of human diseases, including lung cancer.

Finally, high-throughput technologies such as metabolomics, transcriptomics, and epigenomics have been also tested as potential indicators of early lung cancer [100] and the integration of multiple “omics” together with information from medical images and clinical data will provide insightful information for the understanding of human diseases, including lung cancer (Figure 1). In line with this notion, machine learning and deep learning algorithms have been successfully applied to integrate multiple omics, imaging, and clinical data in various research settings [101,102]. Integrating imaging and omics data with the help of deep learning models may help to improve early lung cancer diagnosis. Indeed, recent findings have demonstrated the feasibility of using high-dimensional features derived from CT images of lung cancer that are associated with the presence of specific mutations in tumor tissues by using “radiogenomics”, which refers to the correlation between imaging features and the genomic data obtained from tissue analysis (and other clinical data) to generate imaging surrogates of genetic testing [103]. For example, specific CT scan imaging features have been associated with the presence of tumor driving mutations in lung cancer, including anaplastic lymphoma kinase (ALK), epidermal growth factor receptor (EGFR), Kirsten rat sarcoma viral oncogene homolog (KRAS), rearranged during transfection proto-oncogene (RET) and c-ros oncogene 1 (ROS1) [103,104,105]. Consequently, cancer screening programs will benefit from the AI-based integration, extraction, and interpretation of the vast amount of information generated from these technologies. 

## Figures and Tables

**Table 1:** Summary of articles in which artificial intelligence (AI) was used to analyze low-dose computer tomography (LDCT) images for lung cancer diagnosis.

First Author/Year	Algorithm	Source of Data	No of Cases	Type of Validation	Main Finding
Ciompi F. [66], 2017	CNN: Support vector machines	Data from the MILD trial and DLCST trial	943 patients (1352 nodules) from MILD trial.	468 patients (639 nodules) from DLCST trial. Accuracy: comparing with experienced radiologists (assessing 162 nodules from the test set)	The model outperformed classical patch classification approaches classifying lung nodules and its performance was comparable to that of experienced radiologists
Petousi P [67]	DBNs. Including three expert-driven DBNs and two DBNs derived from structure learning methods.	Retrospective clinical trial data from the NLST Trial.	Trained 10 times. Each time, 400 NLST cases (200 cancer and 200 non-cancer cases) randomly selected.	Internal validation using the complete LDCT arm of the NLST dataset (N = 25,486)	High discrimination and predictive power with the majority of cancer and non-cancer cases. Average AUC of the ROC was >0.75 for all DBN models outperforming logistic regression and naïve Bayes comparison models.
Zhang C [68]	CNN	Chest CT images from 3 sets of data.	888 CT images from the LUNA16 dataset and 1397 CT images from the Kaggle dataset	CT images from three university hospitals in China. 25 experts graded prospectively collected CT images and compared with the CNN model	Performance of the model: Sensitivity 84.4% and specificity 83.0%. Subgroup analysis of smaller nodules (<10 mm) showed high sensitivity and specificity, similar to that of larger nodules (10–30 mm).
Petousi P [69]	Machine learning and DBN	NLST data	5402 cases from the NLST LDCT trial arm with single indeterminate pulmonary nodules	-A five-fold stratified cross validation -Model’s decisions further compared with experts’ decisions	The model lowered the false-positive rate for most screenings in the NLST, while maintaining true positive detection rates; and improved early prediction of cancer cases with indeterminate pulmonary nodules
Huang [70]	Deep learning	Retrospective clinical trial data from the NLST Trial and from PanCan study.	The training cohort: 25,097 NLST cases	Double-blinded validation with 2294 PanCan cases	Compared with Lung-RADS, the model identified a high-risk group that was smaller and had a higher proportion of cancers, and more accurately identified people at very low-risk of lung cancer within 2 years.
Ardila [71], 2019	Deep learning (Three-dimensional deep convolutional neural networks)	Retrospective clinical trial data from the NLST Trial.	6716 NLST cases,	Independent 1139 cases.	AUC, 0.94 Better than radiologists when prior CT was not available. Equal as radiologists when prior CT was available
Cui [72], 2020	Deep learning	Retrospective analysis of lung cancer screening data from three hospitals in China	Training test: 39,014 chest LDCT screening cases	Validation set (600 cases). External validation: the LUNA public database (888 studies)	Higher sensitivity than all radiologists. Low FPR. Better ROC-AUC and higher specificity than radiologists for classifying true positive cases. Good for differentiating nodule dimensions and nodule sub-types.

CNN: convolutional neural network; NR: not reported; AUC: area under the curve; ROC: receiver operating characteristic; DBN: Dynamic Bayesian networks; AUROC: area under the receiver operator characteristic curve; LUNA: Lung Nodule Analysis dataset; LDCT: low-dose computer tomography; MILD: Multicentric Italian Lung Detection; DLCST: Danish Lung Cancer Screening Trial; NLST: National Lung Screening Trial; CT: computer tomography; Lung-RADS: Lung CT Reporting and Data System.

## Supplementary Materials

The following are available online at https://www.mdpi.com/2077-0383/9/12/3860/s1, Table S1: Summary of articles in which AI was used to analyze LDCT images for lung cancer diagnosis.
