# Supplementary material for: Artificial Intelligence Tools for Refining Lung Cancer Screening
Source: J Clin Med. 2020 Nov 27;9(12):3860. doi: 10.3390/jcm9123860 (PMC7760157; doi:10.3390/jcm9123860)
Supplement: Supplementary file 1 [file jcm-09-03860-s001.pdf]

Supplementary Table 1

**Table S1.** Summary of articles in which AI was used to analyze LDCT images for lung cancer diagnosis.

| Study | Algorithm | | | | Model Availability (Open-Access) | Analysis and Additional Features |
|---|---|---|---|---|---|---|
| | Transfer Learning Applied | Algorithm Name Architecture | Algorithm Structure | Model Task | | |
| Ciompi F. [1] 2017 | No | ConvNets<br>CNN<br>"multi-stream multi-scale" architecture | Nine streams of ConvNets, grouped into three sets of three streams.<br>Each set of streams is fed with a triplet of orthogonal patches extracted at the same scale.<br>Optimal architecture for each stream defined using the VGG-net approach | Nodule segmentation, classification, and prediction of nodule type | Yes | SVM to assess intensity features from training set.<br>Features automatically learned from raw data (unsupervised), using the K-means algorithm.<br>Parameters of the network updated using the ADAM algorithm. |
| Petousi P [2] | No | DBN models:<br>-Expert-driven DBNs two Forward-Arrow DBNs and one Reversed-Arrow DBN using a NoisyMax gate<br><br>-Learned DBNs created through structure learning methods. | DBN models<br><br>design process:<br>1-variable selection<br>2-defining the structure (network topology)<br>3-computing the probabilities<br>4-computing the probabilities of the transition model.<br>5-training and testing. | Positive case prediction Both DBN A and B tend to discriminate cancer and non-cancer cases better with increasing number of screenings.<br><br>Comparison with expert radiologists | No | All DBN models were compared with a naïve Bayes model, in which each screening was modeled as independent.<br>The model was trained using the EM algorithm, and tested in Genie.<br>Decision tree model using Rapid Miner (modified C4.5 algorithm) |

| | | | | | | |
|---|---|---|---|---|---|---|
| Zhang C [3] | No | CNN | CNN implemented on the Pytorch platform. A 3-steps model: Preprocessing module, Nodule Cancer Diagnosis Network, and Output Module | Segmentation A 3D pulmonary nodule detection network built to obtain 3D features from the lung images. Calculate image-level malignancy score. | No | PASS 11 software for sample evaluation. Used a two-stage training strategy to increase the stability of CNN learning. Batch normalization and dropout used in the network to improve the training effectiveness and avoid over-fitting |
| Petousi P [4]. | No | ML and DBN | ML and sequential decision-making methods. A framework for learning using a POMDP with the QMDP approximation algorithm. . | Nodule detection and classification. Suggest optimal screening timeline and evaluation metrics. | No | Clinical variables selected in the Tammemägi model. Integrated a DBN into the MDP to predict the chance of developing lung cancer. Applied IRL to formulate a rewards model |
| Huang [5] | Yes | DL | Neural network built to develop the model using an MLP. Included two MLP structures with two hidden layers each. The first had sizes of five and two and the second had sizes of 51 and eight. Weights optimized using a quasi-Newton method and stochastic gradient-based method | Discriminate between benign and malign lesions. Lung cancer risk assessment and cancer incidence prediction at 1 year, 2 years, and 3 years with the Lung-RADS and volume doubling time, using time-dependent AUC analysis. | Yes | Primary analysis compared the lung cancer prediction accuracy among three predictors (DeepLR, Lung-RADS, and VDT). Secondary analysis compared cancer incidence among high-risk and low-risk subgroups. Exploratory survival analyses were done to study |

| | | | | | | |
|---|---|---|---|---|---|---|
| | | | | | | whether the model can detect more aggressive lung cancers. |
| Ardila, [6] 2019 | Yes | Mask RCNN; RetinaNet; Inception V1 | CNN; Inception. The system consists of four components, all trained using the Google Inc. TensorFlow platform: Lung segmentation. Cancer ROI detection model. Full-volume model. Cancer risk prediction model. | The model was trained to take the entire CT volume (the entire set of axial Images) and automatically generated a score predicting whether the patient will have a cancer diagnosis in the same study year | Code not publicly available but some components of this work are available in open source repositories such as Tensorflow, and others | Malignant prediction, nodule localization performed by selecting the ROI with the highest malignancy score |
| Cui [7] 2020 | Yes | 50-layer deep CNN | CNN architecture deep residual network using ResNet | Pulmonary nodule identification. Evaluated diagnostic metrics and agreement between human reviewers and the DL algorithm. | No | Python library and R. Radiologists and algorithm performance assessed using the FROC score, ROC-AUC, and average time consumption |

DL: Deep learning; ML: Machine learning; CNN: convolutional neural network; NR: not reported; AUC: area under the curve; ROC: receiver operating characteristic; DBN: Dynamic Bayesian networks; AUROC: area under the receiver operator characteristic curve; MLP: multilayer perceptron; LUNG-RADS: Lung CT Screening Reporting and Data System; POMDP: Partially observable Markov decision process; IRL: inverse reinforcement learning; SVM: Support Vector Machine; VGG-net: Visual Geometry Group

neural network; ADAM: Adam stochastic optimization algorithm; EM algorithm: expectation–maximization algorithm ; 3D: three-dimensions; CT: computer tomography; VDT: vulnerability detection tools; ROI: tumor region of interest; R: refers to a language and environment for statistical computing and graphics; FROC: Free-Response ROC Curve.

**References**


1. Ciompi, F.; Chung, K.; van Riel, S.J.; Setio, A.A.A.; Gerke, P.K.; Jacobs, C.; Scholten, E.T.; Schaefer-Prokop, C.; Wille, M.M.W.; Marchianò, A., et al. Towards automatic pulmonary nodule management in lung cancer screening with deep learning. *Sci Rep* **2017**, *7*, 46479, doi:10.1038/srep46479.
2. Petousis, P.; Han, S.X.; Aberle, D.; Bui, A.A. Prediction of lung cancer incidence on the low-dose computed tomography arm of the National Lung Screening Trial: A dynamic Bayesian network. *Artif Intell Med* **2016**, *72*, 42-55, doi:10.1016/j.artmed.2016.07.001.
3. Zhang, C.; Sun, X.; Dang, K.; Li, K.; Guo, X.W.; Chang, J.; Yu, Z.Q.; Huang, F.Y.; Wu, Y.S.; Liang, Z., et al. Toward an Expert Level of Lung Cancer Detection and Classification Using a Deep Convolutional Neural Network. *Oncologist* **2019**, *24*, 1159-1165, doi:10.1634/theoncologist.2018-0908.
4. Petousis, P.; Winter, A.; Speier, W.; Aberle, D.R.; Hsu, W.; Bui, A.A.T. Using Sequential Decision Making to Improve Lung Cancer Screening Performance. *IEEE Access* **2019**, *7*, 119403-119419, doi:10.1109/ACCESS.2019.2935763.
5. Huang, P.; Lin, C.T.; Li, Y.; Tammemagi, M.C.; Brock, M.V.; Atkar-Khattra, S.; Xu, Y.; Hu, P.; Mayo, J.R.; Schmidt, H., et al. Prediction of lung cancer risk at follow-up screening with low-dose CT: a training and validation study of a deep learning method. *Lancet Digit Health* **2019**, *1*, e353-e362, doi:10.1016/S2589-7500(19)30159-1.
6. Ardila, D.; Kiraly, A.P.; Bharadwaj, S.; Choi, B.; Reicher, J.J.; Peng, L.; Tse, D.; Etemadi, M.; Ye, W.; Corrado, G., et al. End-to-end lung cancer screening with three-dimensional deep learning on low-dose chest computed tomography. *Nat Med* **2019**, *25*, 954-961, doi:10.1038/s41591-019-0447-x.
7. Cui, S.; Ming, S.; Lin, Y.; Chen, F.; Shen, Q.; Li, H.; Chen, G.; Gong, X.; Wang, H. Development and clinical application of deep learning model for lung nodules screening on CT images. *Sci Rep* **2020**, *10*, 13657, doi:10.1038/s41598-020-70629-3.
